# In vivo efficacy of auranofin in a hamster model of *Clostridioides difficile* infection

**DOI:** 10.1038/s41598-021-86595-3

**Published:** 2021-03-29

**Authors:** Nader S. Abutaleb, Mohamed N. Seleem

**Affiliations:** 1 Department of Comparative Pathobiology, College of Veterinary Medicine, Purdue University, West Lafayette, IN 47907 USA; 2 Department of Biomedical Sciences and Pathobiology, Virginia-Maryland College of Veterinary Medicine, Virginia Polytechnic Institute and State University, 1410 Prices Fork Rd, Blacksburg, VA 24061 USA

**Keywords:** Drug discovery, Microbiology

## Abstract

*Clostridioides difficile* infections (CDIs) are an urgent public health threat worldwide and are a leading cause of morbidity and mortality in healthcare settings. The increasing incidence and severity of infections combined with the scarcity of effective anti-CDI agents has made treatment of CDI very challenging. Therefore, development of new, effective anticlostridial agents remains a high priority. The current study investigated the in vivo efficacy of auranofin in a CDI hamster model. All hamsters treated with auranofin (5 mg/kg) survived a lethal challenge with *C. difficile*. Furthermore, auranofin (5 mg/kg) was as effective as vancomycin, the drug of choice for treatment of CDIs, against relapsing CDI. Furthermore, auranofin (5 mg/kg) generated a 3.15-log_10_ reduction (99.97%) in *C. difficile* count in the cecal contents of hamsters. These results indicate that auranofin warrants further investigation as a new agent to replenish the pipeline of anti-CDI therapeutics.

## Introduction

*Clostridioides difficile* is the most common cause of healthcare-associated infections and antibiotic-associated diarrhea that imposes a heavy burden on most healthcare systems^1,2^. *C. difficile* infection (CDI) is considered a significant source of morbidity and mortality in healthcare settings^3^. According to the U.S. Centers for Disease Control and Prevention (CDC), about 223,900 patients were hospitalized with a CDI in the United States in 2017, which resulted in around 12,800 deaths and over $1 billion in healthcare costs^2^. CDI is considered an urgent threat by the CDC, and controlling CDI is a top priority for healthcare systems to mitigate both clinical and financial outcomes^2^.

In spite of numerous calls for development of new anti-CDI therapeutics, only one new drug, fidaxomicin, has been developed for the treatment of CDIs during the past 30 years^4^. Currently, vancomycin and fidaxomicin are the only drugs approved by the U.S. Food and Drug Administration (FDA) for treatment of CDI. Although previously recommended as a first-line treatment for CDI in adults, metronidazole is no longer recommended for severe CDI cases and is restricted to patients who are unable to obtain or be treated with vancomycin or fidaxomicin^5^. Treatment of CDI currently relies heavily on vancomycin or metronidazole. However, both drugs have drawbacks including high treatment failure and frequent recurrence of disease (in 25–30% of cases)^6,7^. Moreover, both antibiotics induce dysbiosis (disrupting gut microbiota diversity), which enhances susceptibility to CDI^8^. Furthermore, treatment of CDI with vancomycin and metronidazole has been shown to promote the overgrowth of vancomycin-resistant enterococci (VRE)^9^. Fidaxomicin is less damaging to gut microbiota compared to both vancomycin and metronidazole^10^. However, the clinical outcome of fidaxomicin is still unsatisfactory as it pertains to treatment failure, especially in cases of relapsing CDI^11^. Additionally, treatment with fidaxomicin is restricted by its high cost, which is almost 150 times more expensive than metronidazole^12,13^. Emerging resistance or reduced susceptibility to currently available anti-CDI antibiotics has further compounded the challenge to treat CDIs^14,15^. Consequently, there is a critical need to identify and develop new, effective anticlostridial drugs.

De novo drug discovery is a time-consuming and highly expensive process that can take up to 15 years and can cost more than $2 billion to develop one new drug^16^. Drug repurposing is an attractive approach that can lessen the time and cost to develop new therapeutics compared to de novo drug discovery^17–25^. Utilizing a drug repurposing strategy, we identified auranofin, an FDA-approved antirheumaic drug^20,26^, as a potent anticlostridial agent capable of inhibiting production of both toxins and spores in vitro^23^. When investigated in an in vivo mouse model, auranofin (at clinically achievable doses) significantly protected mice from *C. difficile* challenge^24^. Additionally, auranofin significantly prevented CDI recurrence in mice when compared with vancomycin^24^. Building upon our previous studies, the main objectives of the study herein were to investigate the in vivo efficacy of auranofin in a CDI hamster model, establish a dose–response relationship for auranofin in the hamster model of CDI, and to evaluate auranofin’s ability to prevent CDI recurrence.

## Results and discussion

### In vivo efficacy of auranofin in a *C. difficile* ileocecitis hamster model

Auranofin was previously reported to exhibit potent antibacterial and antivirulence activities against *C. difficile *in vitro^23^*.* Additionally, auranofin, at clinically achievable concentrations, was able to protect mice against *C. difficile* challenge^24^. These results encouraged us to investigate auranofin’s efficacy in a *C. difficile* ileocecitis hamster model and auranofin’s potential to protect hamsters from CDI recurrence. The hamster model is routinely used to evaluate therapeutics for treatment of CDI. CDI in hamsters exhibits key morphological features similar to CDI in humans such as colon enlargement, fluid accumulation and pseudomembrane formation. Additionally, dysbiosis induced by clindamycin treatment, which leads to proliferation of *C. difficile*, is observed both in hamsters and in humans^27,28^. In contrast, CDI in hamsters is rapidly fatal if left untreated, a pattern that is not characteristic of human CDI. Thus, the CDI hamster model can be considered as a prevention of death model^29^.

**Figure 1:**
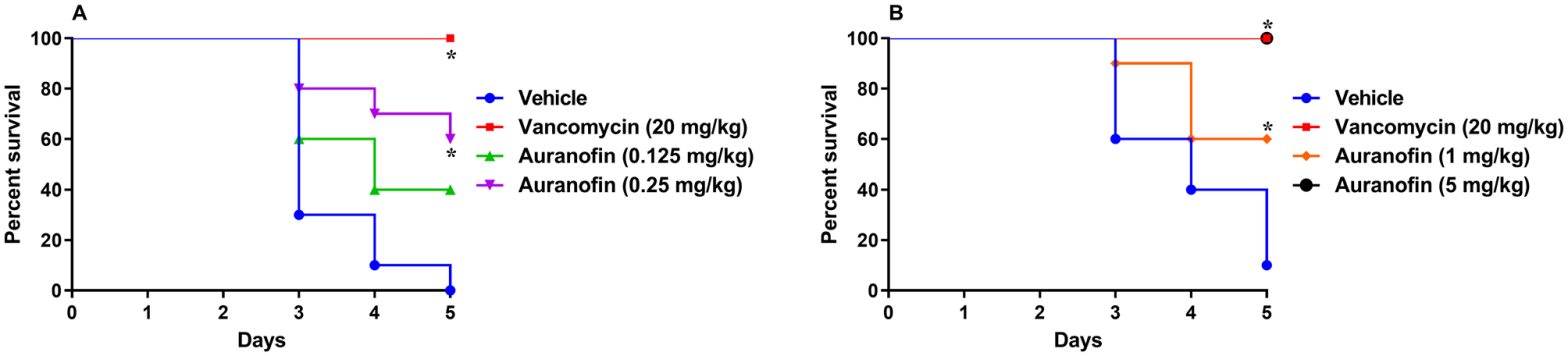
Efficacy of auranofin in treatment of CDI in hamsters: (**A**) low doses of auranofin (0.125 mg/kg and 0.25 mg/kg) and (**B**) high doses of auranofin (1 mg/kg and 5 mg/kg). Hamsters were treated with auranofin, vancomycin (20 mg/kg), or the vehicle for 5 days after infection with *C. difficile* UNT103-1. Kaplan–Meier survival curves were analyzed using a log-rank (Mantel–Cox) and Gehan–Breslow–Wilcoxon tests (P < 0.05). An asterisk (*) denotes a statistically significant difference between hamsters treated with either auranofin or vancomycin in comparison with the vehicle-treated hamsters.

The Golden Syrian hamster model was used to evaluate auranofin’s ability to prevent ileocecitis induced by *C. difficile*, compared to vancomycin. The initial study investigated the activity of low doses of auranofin. Two groups of infected hamsters (n = 10) were treated with 0.125 mg/kg or 0.25 mg/kg of auranofin. One group was treated with vancomycin (positive control) and the last group received the vehicle alone (negative control). Treatments were continued for 5 days during which hamsters were observed for disease symptoms. As shown in Fig. 1A, vehicle-treated hamsters exhibited 100% mortality by day 5 of the study, in agreement with previous reports^30–32^. Vancomycin (20 mg/kg) protected 100% of the infected hamsters up to 5 days, in coincidence with previous reports^30,33,34^. Hamsters administered 0.125 mg/kg of auranofin exhibited 40% survival by day 5. Auranofin, at 0.25 mg/kg, was more efficacious resulting in 60% survival of infected hamsters on day 5, which was statistically significant compared to the vehicle-treated group.

We next tested the effect of higher doses of auranofin (1 mg/kg and 5 mg/kg) (Fig. 1B). After 5 days of auranofin (1 mg/kg) treatment, 60% of hamsters infected with *C. difficile* survived. On the other hand, administration of 5 mg/kg auranofin resulted in 100% survival of infected hamsters during the 5-day treatment period. It is worth mentioning that the results obtained in the in vivo* C. difficile* ileocecitis hamster model were slightly different from the results previously reported in the in vivo CDI mouse model^24^. This effect could be attributed to a difference in auranofin’s pharmacokinetic profile between hamsters and mice. The rates of metabolism and excretion for auranofin may differ between hamsters and mice, which could lead to a difference in the drug’s concentration at the infection site. This factor would need to be further explored in future studies. Another factor that might have contributed to the difference in results obtained between the hamster and mice studies is the overall surface area of the infection site. The site of infection is expected to be larger in hamsters compared to mice. Thus, we suspect that higher doses of auranofin were needed in hamsters to achieve a similar protective effect observed in mice.

**Figure 2:**
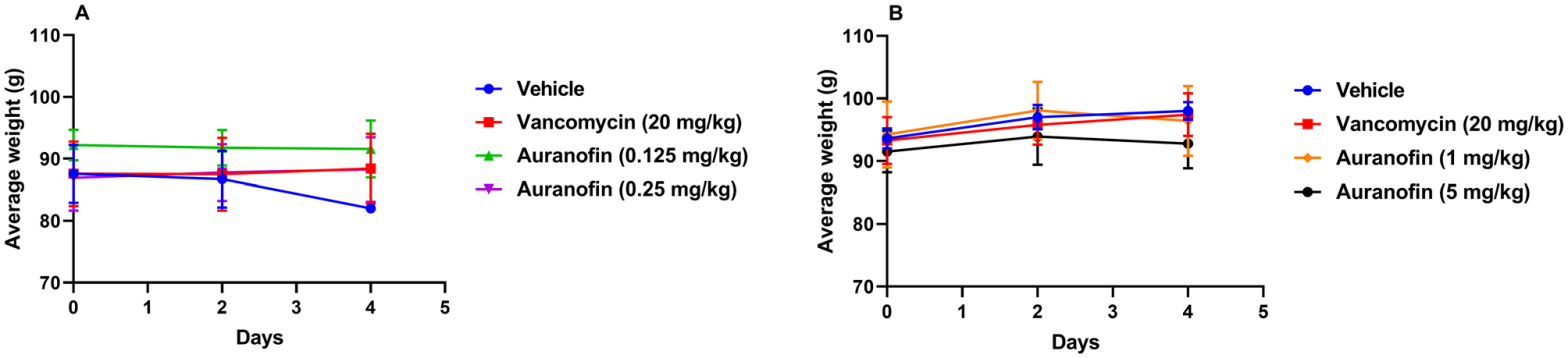
Average weight of surviving hamsters infected with *C. difficile* treated with: (**A**) low doses of auranofin (0.125 mg/kg and 0.25 mg/kg) and (**B**) high doses of auranofin (1 mg/kg and 5 mg/kg). Infected hamsters were treated with auranofin, vancomycin (20 mg/kg), or the vehicle for 5 days and weighed every other day. The data are presented as average weight (g) (mean ± standard deviation) for each group. A two-way ANOVA with post-hoc Dunnett’s test for multiple comparisons (P < 0.05) found no significant difference between the average weight for each group after receiving treatment, as compared to that before the start of treatment (day 0).

During the experiment, the average weight of surviving hamsters in each treatment group was measured every other day (Fig. 2A,B). In the first experiment (Fig. 2A), hamsters in the vehicle-treated group experienced slight weight loss by day 4, but the weight loss was not statistically significant. No decrease in weight was observed for hamsters treated either with auranofin (at 0.125 mg/kg and 0.25 mg/kg) or vancomycin (Fig. 2A). Similarly, in the second experiment, hamsters treated with either auranofin (1 mg/kg and 5 mg/kg) or vancomycin did not exhibit signs of weight loss (Fig. 2B).

### In vivo efficacy of auranofin in a relapsing CDI hamster model

One of the main problems associated with CDI is the high incidence of recurrence (in 15 to 50% of cases) following initial success with antibiotic treatment^35^. Relapsing CDI occurs due to the presence of *C. difficile* spores that germinate in the gut into vegetative cells that colonize the intestine and subsequently produce toxins^36,37^. Recurrence of infection occurs in approximately 20% of patients^15,38,39^. Additionally, 20% of patients who experienced a relapsing episode of *C. difficile* reportedly died within 30 days of diagnosis^40^. Moreover, it was reported that up to 65% of patients successfully treated from CDI recurrence will relapse again in the future^41,42^. Consequently, relapsing CDI represents a difficult and challenging problem facing healthcare systems that requires the discovery of new, more effective agents.

**Figure 3:**
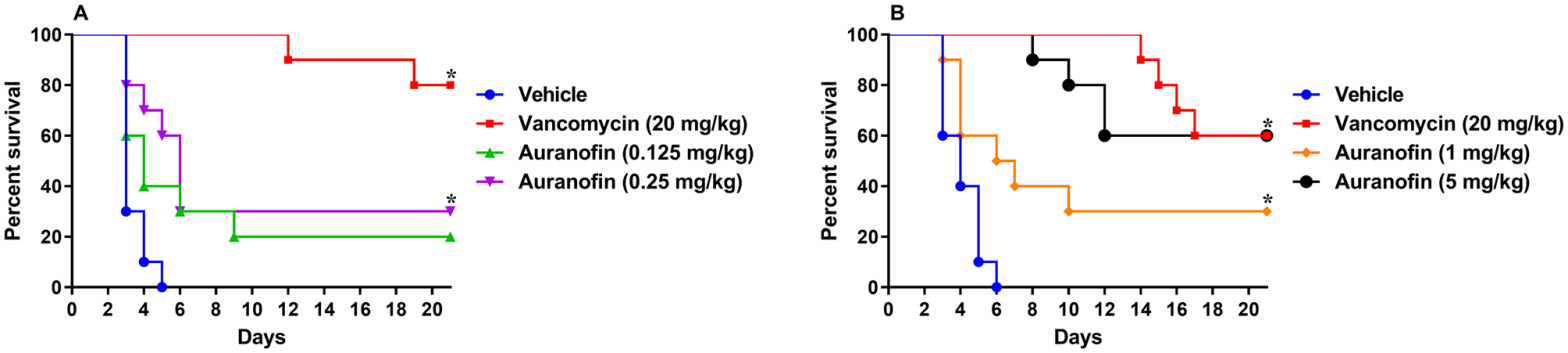
Efficacy of auranofin against CDI recurrence in hamsters: (**A**) low doses (0.125 mg/kg and 0.25 mg/kg) and (**B**) high doses (1 mg/kg and 5 mg/kg). Hamsters were treated with auranofin, vancomycin (20 mg/kg), or the vehicle after infection with *C. difficile* UNT103-1. Treatments were discontinued after 5 days. Kaplan–Meier survival curves were analyzed using a log-rank (Mantel–Cox) and Gehan–Breslow–Wilcoxon tests (P < 0.05). An asterisk (*) denotes a statistically significant difference between hamsters treated with either auranofin or vancomycin in comparison to the vehicle-treated hamsters.

With this issue in mind, we sought to investigate the activity of auranofin in preventing *C. difficile* relapse in hamsters. We initially tested the activity of low doses of auranofin (0.125 mg/kg and 0.25 mg/kg) in a relapsing CDI hamster model (Fig. 3A). This study was followed by another relapsing CDI hamster study investigating the activity of higher auranofin doses (1 mg/kg and 5 mg/kg) (Fig. 3B). In both studies, animals were infected with *C. difficile* and treatments were discontinued after 5 days. Hamsters were subsequently monitored for survival and possible CDI relapse.

As depicted in Fig. 3A, vehicle-treated hamsters became moribund following *C. difficile* challenge resulting in 100% mortality by day 5. This result is in agreement with previous studies^30–32^. Following discontinuation of treatment, vancomycin protected 100% of infected animals through day 12 (90% survival). By day 19, 80% of hamsters in the vancomycin treatment group were alive, which remained unchanged until the end of the experiment. On the other hand, animals administered auranofin (0.25 mg/kg) exhibited a recurrence rate of 50%. Three hamsters died during the post-treatment period resulting in 30% survival by the end of the experiment (statistically significant protection when compared to the vehicle-treated group). Auranofin (0.125 mg/kg) was slightly less efficacious with an overall survival of 20% (50% recurrence rate, as 2 out of 4 hamsters died after the discontinuation of treatment) (Fig. 3A).

We next tested the ability of auranofin at higher doses to prevent CDI recurrence. Two doses of auranofin (1 mg/kg and 5 mg/kg) were evaluated in addition to the vehicle (negative control) and vancomycin (standard-of-care antibiotic). As shown in Fig. 3B, CDI resulted in the mortality of vehicle-treated hamsters with 40%, 60%, 90% and 100% mortality observed on days 3, 4, 5 and 6, respectively. During the post-treatment stage, vancomycin protected all infected animals through day 13. Starting on day 14, a stepwise pattern of mortality was observed ultimately resulting in 60% survival (40% relapse) at the end of the experiment. This pattern is typically observed with vancomycin treatment^30,42^. Auranofin (5 mg/kg) protected all infected hamsters after the discontinuation of treatment through day 8, at which point one hamster died. By day 10, 80% of hamsters treated with auranofin (5 mg/kg) were alive. By day 12, 60% of hamsters were alive, which was maintained until the end of day 21. On the other hand, auranofin (1 mg/kg) was less efficacious with 40% mortality observed during the treatment stage (until day 5). Following the discontinuation of treatment, 3 hamsters succumbed to relapsing CDI, which resulted in 30% survival by the end of the experiment (statistically significant protection as compared to the vehicle-treated group) (Fig. 3B).

**Figure 4:**
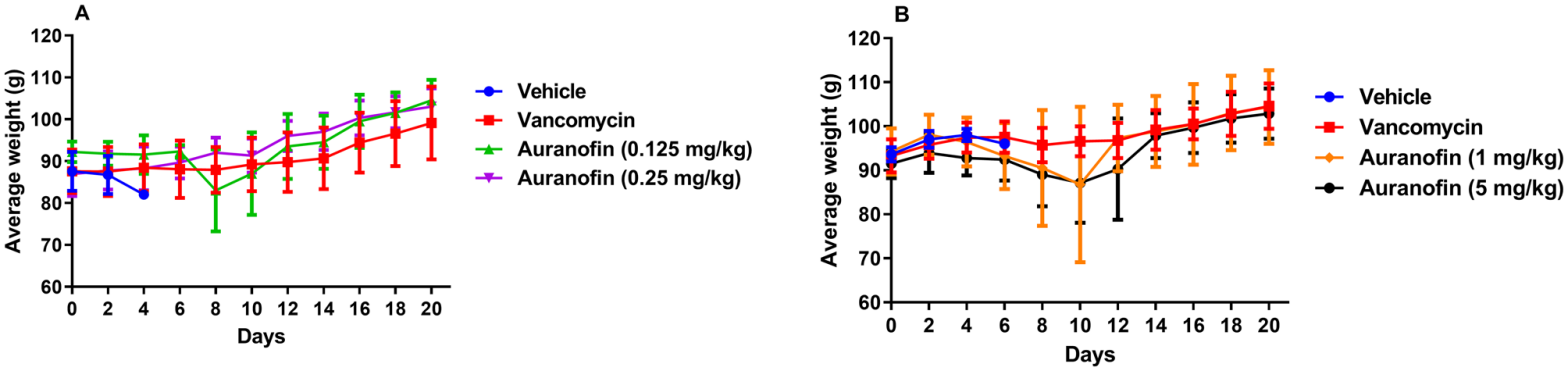
Average weight of all surviving hamsters in the CDI recurrence experiments: (**A**) low doses of auranofin (0.125 mg/kg and 0.25 mg/kg) and (**B**) high doses of auranofin (1 mg/kg and 5 mg/kg). Infected hamsters were treated with auranofin, vancomycin (20 mg/kg), or the vehicle for 5 days and treatments were discontinued thereafter. Hamsters were weighed every other day until the end of each experiment. The data are presented as average weight (g) (mean ± standard deviation) for each group.

Additionally, the average body weight results of the two experiments evaluating low and high doses of auranofin (Fig. 4A,B), found that hamsters treated with auranofin exhibited a slight loss in their average body weight through days 8–10. This was followed by an increase in body weight of hamsters until the end of the study. The initial weight loss was attributed to animals that died later in the study whereas hamsters that survived exhibited an overall increase in average body weight.

After the conclusion of each experiment, hamsters were humanely euthanized and the cecal tissues were aseptically removed, homogenized, diluted and plated to determine the *C. difficile* CFU count inside each hamster’s cecum.

**Figure 5:**
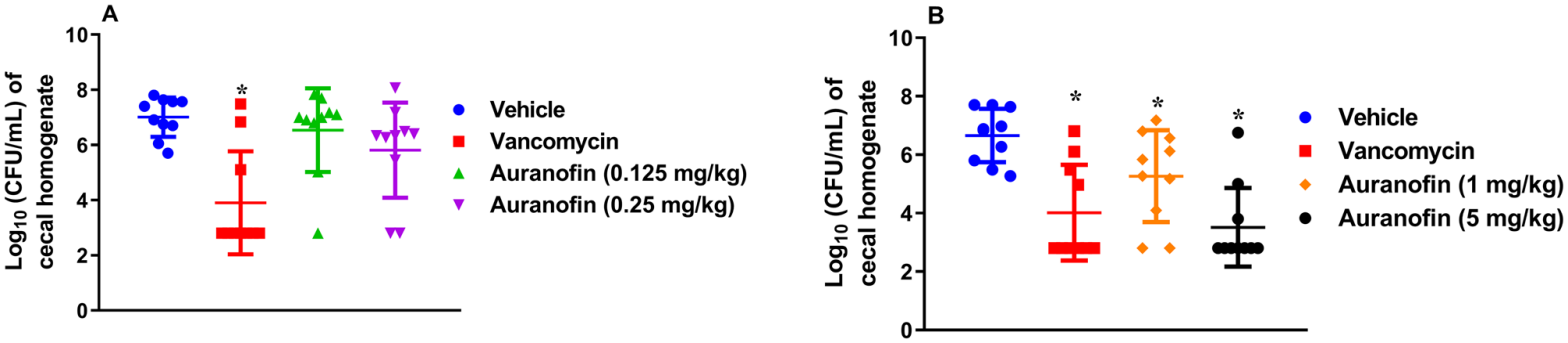
*C. difficile* UNT103-1 CFU counts in the cecal tissues of infected hamsters: (**A**) low doses of auranofin (0.125 mg/kg and 0.25 mg/kg) and (**B**) high doses of auranofin (1 mg/kg and 5 mg/kg). Infected hamsters were treated with auranofin, vancomycin (20 mg/kg), or the vehicle for 5 days and treatments were discontinued thereafter. Bacteria were recovered from the cecal tissues of hamsters under anaerobic aseptic conditions, serially diluted, and plated. The data were analyzed via a one-way ANOVA with post hoc Dunnett's test for multiple comparisons (*P* < 0.05). An asterisk (*) denotes a statistically significant difference between hamsters treated with either auranofin or vancomycin in comparison to the vehicle-treated hamsters.

Low doses of auranofin (0.125 mg/kg and 0.25 mg/kg) were less effective in reducing the *C. difficile* counts inside the cecal tissues generating a 0.48-log_10_ reduction (with 0.125 mg/kg dose) and 1.2-log_10_ reduction (with 0.25 mg/kg dose), respectively (Fig. 5A). Statistical analysis of the data for auranofin (both at 0.125 mg/kg and 0.25 mg/kg) determined that this reduction in bacterial burden was not significant. Notably, two-thirds of hamsters in the auranofin (0.25 mg/kg) group that survived until the end of the study exhibited bacterial CFU counts in the ceca that were below the limit of detection (2.80 log_10_ (CFU/mL)). One hamster in the auranofin (0.125 mg/kg) group also exhibited a CFU count that was below the limit of detection. In contrast, vancomycin significantly reduced the bacterial CFU count by 3.1-log_10_, with 7 hamsters exhibiting bacterial CFU counts in the ceca that were below the limit of detection (Fig. 5A).

In the second experiment, evaluating the activity of higher doses, auranofin (5 mg/kg) was slightly superior to vancomycin in decreasing the burden of *C. difficile* in the cecal tissues of infected hamsters (Fig. 5B). Auranofin (5 mg/kg) significantly reduced the *C. difficile* CFU count generating a 3.15-log_10_ reduction. On the other hand, vancomycin (20 mg/kg) generated a 2.65-log_10_ reduction. It is worth noting that 7 hamsters in the auranofin (5 mg/kg) group and 6 hamsters in the vancomycin group presented with *C. difficile* CFU counts that were below the limit of detection (2.80 log_10_ (CFU/mL). Additionally, auranofin (1 mg/kg) significantly reduced the *C. difficile* CFU count by 1.75-log_10_; 2 hamsters in this group exhibited bacterial CFU counts that were below the limit of detection.

In conclusion, this study investigated the efficacy of auranofin in vivo in a CDI hamster model. Auranofin significantly protected hamsters against lethal CDI when administered at the doses of 1 mg/kg or 5 mg/kg. Furthermore, auranofin (5 mg/kg) was as effective as vancomycin in preventing CDI recurrence in hamsters. Interestingly, auranofin (5 mg/kg) was superior to vancomycin in reducing *C. difficile* counts present in the cecum of infected hamsters. These results indicate that auranofin merits further investigation as a supplement to the dry pipeline of anti-CDI therapeutics. Follow-up studies are warranted to investigate the efficacy of higher doses of auranofin and to evaluate the in vivo activity of auranofin in combination with other anti-CDI drugs.

## Materials and methods

### Media and reagents

Media and reagents were purchased commercially: reinforced clostridial medium (Becton, Dickinson and Company, Cockeysville, MD, USA), phosphate buffered saline (PBS) (Corning, Manassas, VA, USA), vancomycin hydrochloride (Hospira, Lake Forest, IL, USA), oxyrase (Oxyrase Inc, West Mansfield, OH, USA) and clindamycin hydrochloride (TCI America, Portland, OR, USA).

### Preparation of *C. difficile* inoculum for infection of hamsters

*C. difficile* VA11 (UNT103-1) was used to infect hamsters. This bacterial strain is a toxigenic clinical isolate that was responsible for multiple CDI outbreaks in North America^43,44^. The *C. difficile* inoculum used for infection was prepared as described previously^43^. Briefly, resuspended bacterial plates grown onto reinforced clostridial medium + 1% oxyrase were diluted to 1 × 10^7^ CFU/mL. The bacterial inoculum was diluted, plated, and counted on reinforced clostridial medium before being used to infect hamsters.

### In vivo efficacy of auranofin in a CDI ileocecitis hamster model

Both studies were performed as a service provided by the University of North Texas Health Science Center (Fort Worth, TX, USA). The studies are in compliance with the Animal Research: Reporting of In Vivo Experiments (ARRIVE) guidelines. Male Golden Syrian hamsters (weighing 80–100 g) were housed in individually ventilated cages (2 per cage) and received food and water ad libitum. The CDI hamster model was performed as described previously^30,33,34,45^. All hamsters were injected with clindamycin (10 mg/kg) subcutaneously. Twenty-four hours after clindamycin pre-treatment, hamsters were infected via oral gavage with 0.75 mL of the previously prepared *C. difficile* inoculum (~ 7.5 × 10^6^ CFU/hamster)*.* The bacterial inoculum used was re-counted after infection to confirm the infective dose.

Following infection, hamsters were randomly allocated into groups (n = 10) for treatment. Twenty-four hours post-infection, hamsters were treated with low doses of auranofin (0.125 mg/kg and 0.25 mg/kg), vancomycin (20 mg/kg), or the vehicle (10% DMSO in PBS). In a follow-up study, two groups of infected hamsters were treated with higher doses of auranofin (1 mg/kg and 5 mg/kg), one group was treated with vancomycin (20 mg/kg), and one group was vehicle-treated. Treatments were administered orally via oral gavage and continued once daily for 5 days. Hamsters were observed throughout the duration of each experiment for signs of mortality and morbidity, the presence of diarrhea (wet tail), and overall appearance (activity, general response to handling, touch, ruffled fur). Hamsters were weighed every other day. Animals exhibiting moribund state such as prolonged periods of weight loss, prolonged lethargy (> 3 days), paralysis, skin erosions, hunched posture and distended abdomen, were euthanized^43^.

### In vivo efficacy of auranofin in a relapsing CDI hamster model

Hamsters in each experiment were infected, as described above. In the first study, hamsters were treated with either auranofin (0.125 mg/kg), auranofin (0.25 mg/kg), vancomycin (20 mg/kg), or the vehicle for 5 days. In the second study, hamsters were treated with higher doses of auranofin (1 mg/kg and 5 mg/kg), vancomycin (20 mg/kg), and the vehicle for 5 days. Thereafter, treatments were discontinued and hamsters were observed for disease symptoms, recurrence of CDI and signs of mortality and morbidity (described above) until the 21^st^ day. Hamsters judged to be in a moribund state were euthanized. Animals that died during the observation period in each experiment were necropsied. Additionally, the contents of deceased hamsters’ cecal tissues were diluted in PBS and plated anaerobically onto modified reinforced clostridial agar to obtain *C. difficile* CFU counts. After the end of each experiment, surviving hamsters from each experiment were humanely euthanized using CO_2_ asphyxiation. The contents from each hamster’s cecal tissues were diluted in PBS and plated onto modified reinforced clostridial agar to obtain CFU counts.

### Statistical analyses

Kaplan-Meir survival data were analyzed using the log-rank (Mantel-Cox) test and Gehan-Breslow-Wilcoxon test, utilizing GraphPad Prism version 8.0 for Windows (GraphPad Software, La Jolla, CA, USA). The cecal *C. difficile* CFU counts were analyzed via a one-way ANOVA with post hoc Dunnett's test for multiple comparisons (*P* < 0.05).

### Ethical approval

All animal housing and experiments were reviewed, approved and performed under the guidelines of the Institutional Animal Care and Use Committee, University of North Texas Health Science Center and carried out in strict accordance with the recommendations in the Guide for the Care and Use of Laboratory Animals of the National Institutes of Health.

## Data Availability

Data presented in this study are available from the corresponding author upon a proper request.
